# Research Priorities in the Field of Posttraumatic Pain and Disability: Results of a Transdisciplinary Consensus-Generating Workshop

**DOI:** 10.1155/2016/1859434

**Published:** 2016-06-14

**Authors:** David M. Walton, James M. Elliott, Joshua Lee, Eldon Loh, Joy C. MacDermid, Siobhan Schabrun, Walter L. Siqueira, Brian D. Corneil, Bill Aal, Trevor Birmingham, Amy Brown, Lynn K. Cooper, James P. Dickey, S. Jeffrey Dixon, Douglas D. Fraser, Joseph S. Gati, Gregory B. Gloor, Gordon Good, David Holdsworth, Samuel A. McLean, Wanda Millard, Jordan Miller, Jackie Sadi, David A. Seminowicz, J. Kevin Shoemaker, Gunter P. Siegmund, Theodore Vertseegh, Timothy H. Wideman

**Affiliations:** ^1^Western University, London, ON, Canada N6G 1H1; ^2^Northwestern University, Chicago, IL 60611, USA; ^3^McMaster University, Hamilton, ON, Canada L8S 4K1; ^4^University of Western Sydney, Campbelltown, NSW 2753, Australia; ^5^University of Washington, Seattle, WA 98195, USA; ^6^Canadian Chiropractic Association, Toronto, ON, Canada M5T 3B2; ^7^Canadian Pain Coalition, Oshawa, ON, Canada L1J 8P7; ^8^Good Law Office, London, ON, Canada N6A 5J7; ^9^University of North Carolina, Chapel Hill, NC 27599, USA; ^10^University of Maryland, College Park, MD 21201, USA; ^11^University of British Columbia, Vancouver, BC, Canada V7A 4S5; ^12^McGill University, Montreal, QC, Canada H3G 1Y5

## Abstract

*Background*. Chronic or persistent pain and disability following noncatastrophic “musculoskeletal” (MSK) trauma is a pervasive public health problem. Recent intervention trials have provided little evidence of benefit from several specific treatments for preventing chronic problems. Such findings may appear to argue against formal targeted intervention for MSK traumas. However, these negative findings may reflect a lack of understanding of the causal mechanisms underlying the transition from acute to chronic pain, rendering informed and objective treatment decisions difficult. The Canadian Institutes of Health Research (CIHR) Institute of Musculoskeletal Health and Arthritis (IMHA) has recently identified better understanding of causal mechanisms as one of three priority foci of their most recent strategic plan.* Objectives*. A 2-day invitation-only active participation workshop was held in March 2015 that included 30 academics, clinicians, and consumers with the purpose of identifying consensus research priorities in the field of trauma-related MSK pain and disability, prediction, and prevention.* Methods*. Conversations were recorded, explored thematically, and member-checked for accuracy.* Results*. From the discussions, 13 themes were generated that ranged from a focus on identifying causal mechanisms and models to challenges with funding and patient engagement.* Discussion*. Novel priorities included the inclusion of consumer groups in research from the early conceptualization and design stages and interdisciplinary longitudinal studies that include evaluation of integrated phenotypes and mechanisms.

## 1. Introduction

Millions of musculoskeletal (MSK) injuries occur daily in diverse settings, ranging from those that are highly distressing (e.g., war, crime, and natural disasters) to those that are relatively benign (e.g., household chores and recreation). Despite these different contexts, many individuals with injuries classified as “noncatastrophic” or “soft-tissue” type disorders will develop persistent pain or other symptoms that can rarely be attributed to obvious structural lesions on diagnostic imaging [1]. For some individuals, these persistent problems are severe and debilitating [2, 3]. Index examples include (i) whiplash-associated disorder (WAD) developing after motor vehicle collision and (ii) occupational low back pain (oLBP), both common and costly conditions for which an estimated 20–50% of individuals report persistent symptoms or interference in daily function 12 months later [3, 4]. There are currently no objective markers of disease severity for these conditions. Treatments are costly, often have limited effectiveness, and may have their own risks. For example, Canadians are the world's largest per capita consumers of opioid and nonsteroidal anti-inflammatory medication for chronic pain [5], and opioid misuse continues to emerge as a public health issue. This raises competing public health concerns: poorly or unmanaged chronic pain and rising concern over opioid misuse and abuse.

Many experts have suggested that the best method to address the epidemic of chronic pain and the high rate of chronicity following acute injury is to identify pathophysiologic mechanisms in the acute stage of injury and intervene accordingly [6, 7]. The questions of who develops persistent (chronic) problems and why these conditions develop have vexed clinicians, policy makers, consumers, and researchers for decades. The pool of scientific and pseudoscientific literature reveals a wide range of models that attempt to explain the transition from acute to persistent problems (pain and disability). These models range from purely biological [8] or psychological explanations [9], to conceptualizing chronic pain as a socially constructed phenomenon resulting from overly solicitous financial compensation paradigms and secondary gain [10, 11]. Some integrated models have been published over the previous decade [12–14] that begin to incorporate the complexity of the problem of symptom persistence related to physical trauma.

The exploration and development of clinical tools and prediction rules have provided an opportunity for clinicians to confidently stratify acutely injured patients by level of risk (low, moderate, and high) [15–17]. While useful in some contexts, many such tools include nonmodifiable factors (e.g., age and gender) that have value for stratification but less obvious value for treatment planning. Additionally, many have yet to be validated in independent populations and/or have not achieved widespread uptake and implementation. Even where risk can be well stratified, targeted treatment has yet to consistently prevent chronicity when compared to standard care in conditions such as traumatic neck pain [18, 19]. One interpretation of these findings is that active or formal rehabilitation for conditions such as acute WAD or oLBP is ineffective. However, many of these studies have identified risk factors using correlational analysis, which reveals association but not necessarily causation. Novel approaches are likely required to reduce the rate of chronicity including focusing research on causal mechanisms that could reveal novel and effective interventions for preventing the transition from acute to chronic pain.

The Institute of Musculoskeletal Health and Arthritis (IMHA), one of the Canadian Institutes of Health Research (CIHR), recognized this need in its 2014–2018 Strategic Plan that describes three areas of priority: (i) chronic pain and fatigue; (ii) inflammation and tissue repair; and (iii) disability, mobility, and health [20]. Explicit subfoci of relevance to the report herein include “*developing a better understanding of the complex causes and clinical manifestations of chronic pain and fatigue*” and “*prevention of chronic musculoskeletal, skin, and oral health disorders through the identification and management of common risk factors*.” IMHA recognizes that rigorous, collaborative, transdisciplinary research is required to address these foci and that integrating research into practice must be a priority. These strategic directions guided a 2-day interactive CIHR-funded workshop held at Western University in London, Ontario, Canada, in March 2015 with the theme of “developing a stakeholder-driven research and knowledge exchange agenda to improve outcomes of injury-related pain and disability.” This workshop brought together researchers, clinicians, and consumers to develop integrated, transdisciplinary research priorities in this field. The purpose of this paper is to report and thematically describe the outcomes and research priorities generated at that meeting.

## 2. Participants

Workshop participants (*N* = 25 in attendance and input from 5 additional individuals who were not able to attend in person) were (i) recognized academic experts in the field of trauma-related pain and injury; (ii) experts in fields not traditionally associated with trauma-related pain and disability but that held potential for expanding the breadth of trauma research; or (iii) consumer group opinion leaders. Academic backgrounds of participants included biochemistry, microbiology, proteomics, biomechanics, kinesiology, imaging physics, neurophysiology, neuroimaging, rehabilitation, genomics, pharmacology, psychology, measurement, traumatology, and epidemiology. Knowledge-user representation included physical medicine, emergency medicine, physical therapy, chiropractic, personal injury law, and patient advocacy. Geographic representation was from the Canadian provinces Ontario, Quebec, British Columbia, and Alberta as well as the United States (North Carolina, Illinois, and Maryland) and Australia (New South Wales state).

## 3. Structure of the Meeting

The meeting consisted of six 1.5-hour blocks. Each block consisted of 4 short “TED”-type talks (5 minutes each), followed by 45 minutes of small group discussion (4–8 participants each) focused on potential points of convergence emerging from the short talks, integration, and collaboration opportunities and barriers, with a subsequent 25-minute large group discussion to “harvest” key topics identified by the small groups. A professional facilitator with no stake in the outcome ensured that the sessions kept to time were productive and that each participant had an adequate voice. A seventh large group discussion was reserved for identifying emergent themes, short- and long-term priorities, and champions for each priority.

## 4. Sponsors and Roles

The event was sponsored by CIHR, the Orthopedic Division of the Canadian Physiotherapy Association (OD-CPA), the London Orthopedic Unit (LOU, a unit of the OD-CPA), Gordon Good Law Offices, the Cluster of Research Excellence in Cognitive Neuroscience at Western University, the Cluster of Research Excellence in Musculoskeletal Health at Western University, the Schulich School of Medicine & Dentistry, and the Faculty of Health Sciences at Western University. With the exception of CIHR, each sponsor had representation at the workshop, but sponsorship did not depend on the outcomes. Participant travel and meals, where required, were paid through the funders and sponsors of this event, as were the professional facilitator, room rental, and a team-building event.

## 5. Emergent Themes and Priorities

Ideas were generated and key conversation points were hand-recorded in each small breakout group. Following completion of the 2-day workshop, all recordings were collected and collated, and a thematic analysis was conducted by two authors following an established protocol [21]. The emergent themes were subsequently vetted by a core authorship team of 6 and then member-checked for accuracy and trustworthiness by all members of the workshop and authorship team. A total of 13 unique themes were identified and described and summarized as follows.

*Emergent Themes Arising from Thematic Analysis of Recordings of 2-Day Active Participation Workshop*. Consider the following:

1. The complex nature of acute and chronic pain.
2. Cause-and-effect longitudinal modeling rather than correlation.
3. Recovery starting immediately following injury.
4. Biological markers, from broad to focused using novel experimental pain paradigms in humans and animals.
5. Leveraging new technologies to solve old problems.
6. Judicious dissemination: the consumer perspective of new research findings.
7. Broad, population-based longitudinal cohort research starting from the premorbid period.
8. Respecting context.
9. The need for integration and convergence of knowledge and direction across disciplines.
10. Meaningful outcomes.
11. Struggles with patient recruitment and engagement.
12. Public awareness and shifting paradigms.
13. Sources of funding for research in posttrauma pain and disability.

*(1) The Complex Nature of Acute and Chronic Pain*. All participants agreed that reducing the experience of acute or chronic pain to purely anatomical or biomechanical causes was overly simplistic and ineffective. Pain is a multifactor experience, with established models describing biological, psychological, and socioenvironmental influences. Disability, defined here as a limitation in person-specific normal daily functioning, is a separate but related construct that requires alternative modes of exploration. The experience of trauma itself, with or without tissue damage, leads to a cascade of cellular processes that, under ideal conditions, are highly coordinated and interdependent. These include activation of ATP/purinergic signaling, locus coeruleus/norepinephrine pathways, immune signaling, and the hypothalamic-pituitary-adrenal axis among many others. Activity in these pathways is not consistent across individuals and is influenced by genetic and transcriptomic profiles, the microbial environment, nutrition, psychological distress, and the social environment. Despite this complexity, participants were confident that key pathways and processes could be identified and pointed to the human genome (https://www.genome.gov/), proteome (http://www.thehpp.org/), and metabolome (http://www.hmdb.ca/) projects as evidence that complex systems biology can be successfully quantified and understood when the required resources are committed. Suggestions for future priorities included (i) integration of psychological and biomarker quantification within biomechanics research and (ii) genomic, transcriptomic, proteomic, and metabolomic (omics) research that can now be conducted with relatively low burden to the patient (e.g., using blood and/or salivary samples).

*(2) Cause-and-Effect and Longitudinal Modeling rather than Correlation*. Risk stratification and identification research is dominated by largely correlational analyses showing an association between a variable collected in the acute stage of injury and outcomes measured 3, 6, or 12 months later. Few, if any, of these associations can be confidently considered truly causative. Certain demographic variables, such as female sex, older age, and lower socioeconomic status (SES), have demonstrated consistent associations with outcome [22]. However, an important question is as follows: does being female or of a low SES status* cause* chronic pain? The participants largely agreed that there are likely independent, overlapping, proxy, mediating, or moderating variables present that have either not been captured in such research or have not been adequately modeled. Understanding the precise mechanisms responsible for identified associations will result in the identification of more meaningful, and potentially modifiable, causes of chronic problems.

The group was largely in agreement with Bradford-Hill's criteria for cause-and-effect [23]: strength, consistency, specificity, temporality, dose-response, plausibility, coherence, and the newer addition of reversibility. A recognized challenge in proving cause-and-effect is that, in the vast majority of research, patient status is unknown* prior* to the event. Indeed, in many such cohort studies, inception occurs within some time frame following an injurious event, ranging from hours to 4 weeks or longer [24–27]. The group agreed that large population-based cohort studies that provide data prior to exposure to the event (trauma) are required to establish the causal and temporal characteristics of changes in biology or psychology. Regular and frequent postevent data capture is important to fully explore the temporal and causal influences of biological and psychological changes on the recovery status of the individual. New technology that permits rigorous assay of biomarkers from body fluids such as saliva and urine could mitigate the traditional burden of repeated blood draws. Understanding cause-and-effect is also critical to establishing causation in personal injury proceedings, wherein the plaintiff must show that, but for the event (injury), they would not have chronic pain.

*(3) Recovery Starts Immediately following Injury*. The participants, especially front-line clinicians who routinely receive and stabilize acute patients, appreciated the importance of their treatment in the subsequent genesis of persistent pain or disability. Reactions to trauma, including stress and inflammatory and psychological responses, are immediate and should therefore be included as part of acute posttrauma evaluation. Participants heard that the wording used, behavior demonstrated, and messages delivered by the acute trauma team (including first responders and emergency room staff) “set the stage” for the postinjury trajectory. Participants were sensitive to the effects (biological and psychological) of spending hours immobilized on a spinal board while waiting for the clinician, undergoing multiple diagnostic tests, and well-meaning but potentially misinterpreted advice given to a patient at the point of acute care. To this end, the participants identified a priority area around more informed management of acutely injured patients through translatable research and efficient clinical pathways.

*(4) Biological Markers*—*from Broad to Focused Using Novel Experimental Pain Paradigms in Humans and Animals*. Although “omics” explorations are novel and emerging, the team recognized the need to balance broad association studies with hypothesis-driven research. While genomic approaches have provided some consistent findings in the chronic pain transition literature (e.g.,* COMT, FKBP5* predicting outcomes of whiplash [28, 29]), the fields of proteomics (the large-scale study of proteins including their presence, concentration, structure, and interactions), transcriptomics (the large-scale study of RNA transcripts (transcriptomes) resulting from gene expression and transcoding), and metabolomics (the large-scale study of chemical processes through evaluation of metabolites resulting from chemical reactions) are still emerging and there has been no empirical evidence of predictive markers for acute to chronic pain transition. One potential approach to mitigating the need for very large samples in wide association studies was proposed through use of novel animal or human models and experimental pain or trauma protocols. An example is intramuscular nerve growth factor (NGF) that has shown promise as a lab-based model for chronic pain [30] and may help to narrow the scope of research prior to moving to longitudinal patient populations. In addition to biochemical or biomechanical marker quantification, functional neuroimaging or motor/reflex (Transcranial Magnetic Stimulation) and sensory (electroencephalography and functional MRI) mapping before, during, and after experimental pain protocols was proposed as a potential approach to integrating cellular and neurophysiology research lines.

*(5) Leveraging New Technologies to Solve Old Problems*. New and advancing technologies are offering the ability for rigorous data collection with unique contrast and better resolution than previous attempts while minimizing patient burden. Clinical MRI systems at field strengths of 3 T are commonplace in most tertiary centres while the emergence of ultrahigh field MRI at 7 T is becoming available for research investigations and is providing considerably improved resolution and novel contrast for both neuroimaging and MSK applications. Recent reports using novel applications of Magnetization Transfer Ratios (MTR) and Magnetic Resonance Spectroscopy (MRS) have revealed new mechanisms of posttrauma pain, including subclinical spinal tract damage and muscle fatty infiltration [31, 32]. Nuclear Magnetic Resonance Spectroscopy (NMRS) acquisitions and editing techniques have been developed that can quantify nearly 70 metabolites from a single blood draw and can do so while preserving the sample for additional assays [33]. Mass spectrometry protocols have been developed for salivary proteomic analysis that can at least theoretically identify over 3000 serum proteins in a 100 *μ*L sample of saliva [34]. Hair is proving to be a useful marker for preinjury status, storing several hormones and peptides, most notably cortisol, in such a way that a retrospective “calendar” of hypothalamic-pituitary-adrenal (HPA) axis activity can be constructed and compared to postinjury status [35, 36]. This approach offers an intriguing opportunity for retrospective longitudinal modeling. Of particular interest is that these new technologies quantify physiological processes with relatively low burden, using non- or minimally invasive procedures or imaging protocols that can be performed in a fraction of the time required for available measures just 5 years ago [37]. These efficiencies now offer the potential for more frequent and less costly repeated measurement for robust longitudinal modeling.

*(6) Judicious Dissemination*—*The Consumer Perspective of New Research Findings*. Well-intentioned and useful new knowledge can lead to problems if not translated and disseminated prudently. The group was reminded that research findings describing primarily psychological causes of chronic pain (e.g., irrational fear or catastrophization) can add stigma to a patient population that already struggles for validation. As well, neuroimaging research, which has propelled the field of chronic MSK pain rapidly forward, can negatively affect a patient's sense of self-control and security. Rote reporting of findings such as “reduced cortical thickness” or “advanced brain aging” may implicitly or explicitly attempt to generalize from patients with longstanding severe pain and disability from tertiary care clinics to all patients with pain. Doing so can easily lead to considerable distress in consumer populations if not presented with appropriate interpretation. In this light, the group agreed that consumer participation at all stages of research, from conceptualization through interpretation and dissemination of results, should be an important priority in the field.

*(7) Broad, Population-Based Longitudinal Cohort Research Starting from the Premorbid Period*. The vast majority of prospective research on traumatic pain enrolled patients* after* the inciting event and, therefore, it is difficult to establish changes in key biological or psychological markers. Participants pointed to the Framingham study [38] and related population-based cohorts as a valuable but costly approach. An alternative to a full population-wide cohort study involves smaller subpopulations who are at high risk of injury and allow enrolment and data collection to occur prior to injury. Examples included sports teams, motor vehicle insurance cohorts, those working in high-risk occupations, or surgical cohorts. In all cases, the potential exists for a rapid change in health status, from healthy to injured, or in the case of surgery, from one health-affected status to a different health-affected status. Where full population-based studies are not feasible, these at-risk subpopulations may represent a useful place to start.

*(8) Respecting Context*. Injury does not occur in a vacuum; rather, a host of social influences interact with the biological and psychological effects of trauma to create the overall “response to trauma.” Participants identified a research priority around identifying social or contextual influences on the experience of pain and disability after trauma. Examples of such influences include access to and availability of care; the compensation/litigation environment; waiting times to be seen by a healthcare provider; waiting times to see medical specialists; adversarial interactions with the other party in the case of two-party trauma; workplace environment and availability of accommodations; and support from spouses, employers, colleagues, coaches, insurers, health providers, or others. Other more macrolevel influences, including prevailing cultural beliefs about injury outcomes, geographically or culturally influenced nutrition, physical activity, health promotion/prevention practices, and geographically determined weather and accessibility issues, could also influence outcomes. The status of such variables as either direct causative factors or mediators or moderators of other variables in the transition from acute to chronic pain is a particular priority for future studies.

*(9) The Need for Integration and Convergence of Knowledge and Direction across Disciplines*. Research in trauma, pain, and disability is advancing rapidly and in many directions. Several aspects of system function— biological and psychological—are currently being explored, but rarely in the same cohort. Anecdotally, some participants described interactions with large public funding bodies who expressed disinterest in supporting intervention trials for conditions such as acute WAD or oLBP until the mechanisms underlying these conditions and their transition to chronicity are clarified. Therefore, a priority is to actively establish productive transdisciplinary research teams with members from a breadth of academic disciplines. In this way, both basic and clinical research can be conducted simultaneously and in the same patient population, offering the potential to link biological parameters with clinical signs and symptoms. Aligning an entire field of research may not be feasible and risks stifling innovation. On the other hand, having a general field-wide direction with desired outcomes such as improved quality of life or faster symptom resolution would foster cross-fertilization. As well, it would encourage basic scientists to consider the long-term clinical translatability of their findings and clinical scientists to consider potential mechanistic explanations for their clinical observations.

*(10) Meaningful Outcomes*. Acute and chronic pain and disability are nebulous constructs that require valid and meaningful outcomes that can be applied to both research and clinical practice. The IMMPACT group [39] has proposed and aligned outcomes for pain clinical trials, but longitudinal (observational) research has yet to catch up. The definition of a “good” or “bad” outcome following trauma exposure is context-dependent and is likely overly simplistic. Most clinical risk stratification tools recognize a large middle ground for risk of chronicity, suggesting that outcomes need to be graded more finely than just “recovered” or “not recovered” with respect to pain, disability, or occupational engagement. Workshop participants recognized the need to include measurement and qualitative scientists in these discussions, and recommended refining existing outcomes or developing new ones that are meaningful and translatable between the lab and clinic as a high priority area. This was also identified as an area where consumers may be able to play an important role.

*(11) Struggles with Patient Recruitment and Engagement*. Some workshop participants described the difficulty of enrolling patients with acute MSK injuries into research studies focused on long-term outcomes. It was largely recognized by both academics and patient advocates that many people with chronic pain are intrinsically motivated to participate in studies that may lead to a change in their condition. However, it is inappropriate to assume that those with acute injuries possess the same level of intrinsic motivation or engagement in research. Subject attrition or “loss to follow-up” was also identified as a challenge to successful longitudinal studies. The group recognized the need to “sell” the value of such research to those with acute injuries and that effective strategies for retaining subjects include appropriate compensation and frequent contact [40].

Treating clinicians, often the first point of contact for injured patients, were identified as either a facilitator or barrier to recruitment. Emergency medicine is correctly and necessarily focused on ensuring that patients are stabilized and out of immediate threat. This busy environment leaves little time for identification and enrollment of potential study participants. However, this environment is also key for setting up smooth recovery; hence these clinicians are important stakeholders in posttrauma rehabilitation and recovery. Engaging clinicians in research design and respecting their time spent recruiting patients for research are additional priorities.

Finally, it was proposed that injured individuals involved in compensation or litigation related to their injuries appear to be less receptive to research participation. Whether this is due to cynicism/skepticism (e.g., “how might my data be used against me?”) or the added time and stress of active litigation, this barrier to recruitment represents a potential bias of* assembly* in research design. Although not a fatal threat to internal validity, such assembly biases should be recognized in publications arising from such research, which should include the limitations of generalizing the findings between litigating and nonlitigating cohorts.

*(12) Public Awareness and Shifting Paradigms*. Patient recruitment and engagement may also be linked to cultural beliefs or access to care, both of which may also affect the transition to recovery/chronicity. The group identified that public education campaigns are a valuable priority for addressing this factor and pointed to success of such awareness campaigns in regions like Queensland, Australia [41]. A well-orchestrated public education campaign, such as that recently implemented successfully by mental health advocacy groups in Canada (e.g., the “*Depression Hurts*” campaign, http://www.depressionhurts.ca/), may have value as an intervention strategy. Even before employing such practices for prevention or intervention, workshop participants identified public awareness of the magnitude of the chronic pain problem as a potential tool for facilitating engagement. Print and social media were acknowledged as potential avenues for promotion and publicity as were links with existing advocacy and/or special interest groups such as the Canadian Pain Coalition or Canadian/American Pain Societies that have experience and success in large public education campaigns.

*(13) Sources of Funding for Research in Posttrauma Pain and Disability*. It has been reported that public funding for clinical health research, especially in the field of MSK pain, has been stable or decreasing in recent years in Canada [42]. In contrast, the priorities identified herein demand large financial resources for successful implementation. The group discussed the value of private or industry sponsorship and identified potential tensions between the need for financial support to conduct this research and the potential for partners to influence interpretation or dissemination of results. Therefore, a priority recommendation was to work through university-industry liaison offices to establish collegial relationships with potential sponsors that would allow meaningful research to be conducted as described above with memorandums of understanding that dissemination would not be impeded by those sponsors. A good first step would be to partner with industry to establish biobanking infrastructure, allowing storage of biological samples and data that could subsequently be used to leverage public funds for more extensive research programs.

## 6. Discussion and Conclusions

This paper reports the results of a 2-day interactive workshop informed by thirty academics, clinicians, and consumer representatives to establish a series of consensus priorities in the field of “soft-tissue”-type acute and chronic MSK trauma-related pain and disability. In total, 13 priority themes emerged that ranged from issues around funding and patient engagement to evaluating cause-and-effect at both the micro- and macrolevels. All participants, regardless of field or discipline, agreed that simple bivariate correlational research in a single domain needed to be phased out in favour of transdisciplinary explanatory modeling using a mix of hypothesis-generating and hypothesis-driven strategies.

The concept of molecular and neurobiological studies, from preinjury through to several postinjury periods, was a consistent theme that was endorsed as a highly promising way forward in the search for causal mechanisms. However, such work requires considerable financial and infrastructure resources and, therefore, represents a long-term priority. Consequently, other shorter-term priorities were generated that endorsed the use of animal models or novel experimental pain protocols in humans; these offer the potential for more focused measurement and quantification in a larger prospective study. New and advancing technologies mean that a wide spectrum of genomic, transcriptomic, proteomic, and metabolomic factors through to systems-based neural, endocrine, and biomechanical factors can be captured feasibly in a short period of time using non- or minimally invasive techniques.

The contrasting approaches between “fishing” for associations and the need for innovative exploratory research were recognized and discussed. For example, we identified that* hypothesis-driven* research is appropriate for areas where current evidence is clearly pointing towards specific physiologic systems (e.g., catecholaminergic, purinergic, and hypothalamic-pituitary-adrenal). In contrast, more* hypothesis-generating* approaches related to identifying potential mediators/moderators of chronic pain development are appropriate where current evidence is insufficient and/or additional factors and mechanisms are being sought.

Regardless of their current academic or clinical alignments, each participant endorsed research in this field as a high priority. Concern was apparent over rehabilitation funding paradigms for both clinical practice and research that appear to be diminishing based on inconsistent clinical outcomes and a handful of well-designed clinical trials showing little or no effectiveness of active rehabilitation for preventing chronic problems such as WAD [18, 19]. There continue to be large knowledge gaps in the field, from basic causal mechanisms through to appropriate outcomes, such that the success of a trial based on contemporary knowledge may be more a case of extreme good luck rather than evidence for or against the intervention.

This report represents a summarized, factual description of targeted priorities, generating meeting, and as such limitations to interpretation are largely with respect to the perspectives of the invitees and the authors responsible for thematically summarizing the discussion. It is recognized that not all relevant disciplines could logically be invited; notably there was little or no representation from fields such as occupational health or vocational rehabilitation. This limitation will be addressed at a planned 2-year follow-up meeting to review and revise these initial priorities.

The results of this workshop are a transdisciplinary research agenda focused on identifying the cause(s), prevention, diagnosis, prognosis, and cure for chronic pain following injury. Hopefully, this agenda will facilitate the allocation of research resources to areas of high yield and impact. Given IMHA's expressed focus on improving understanding of mechanisms to explain the transition from acute to chronic pain and continued pressures on medical and rehabilitation funding for many MSK injuries, the priorities identified by this workshop appear timely and feasible with recent advances in technology and growing public awareness. Improved outcomes, reduced cost, and decreased burden of chronic pain for the individual and society are goals that we believe all stakeholders can endorse.
